# Humour-driven risk communication and community engagement to mitigate dengue: Lao People's Democratic Republic, 2024

**DOI:** 10.5365/wpsar.2026.17.2.1372

**Published:** 2026-06-01

**Authors:** Visith Khamlusa, Vilakone Phangkhamhack, William Seal, Outhikone Souphome In, Rachel Lorimer, Patrice Suyot, Lieke Visser

**Affiliations:** a Ministry of Health, Vientiane, Lao People’s Democratic Republic.; b World Health Organization Representative Office for the Lao People’s Democratic Republic, Vientiane, Lao People’s Democratic Republic.; c United Nations Foundation, Washington, DC, United States of America.; d World Health Organization Regional Office for the Western Pacific, Manila, Philippines.

## Abstract

**Problem:**

In early 2024, Lao People's Democratic Republic experienced a surge in dengue cases approaching levels of its 2013 epidemic, underscoring the persistent threat of dengue during the monsoon season. Although awareness of preventive behaviours is high, inconsistent adoption has limited their effectiveness.

**Context:**

In response, the Ministry of Health, in collaboration with the World Health Organization, implemented an innovative, humour-driven risk communication and community engagement (RCCE) campaign targeting individuals who perceived dengue as a routine health concern.

**Action:**

To capture attention and increase message retention, the April to September campaign employed an actor dressed as a giant mosquito who deliberately and humorously interrupted daily activities. The strategy used pre-tested materials, a strong social media presence, and television and radio to promote mosquito control, symptom recognition and timely medical care for severe dengue. The impact was assessed through nationwide phone and online surveys and social media analytics.

**Outcome:**

The campaign reached almost 2.9 million unique users (68.2% of whom were using the country’s most popular social media platform), generating more than 27 million content views and 86 342 interactions. Post-campaign surveys indicated a sharp rise in public concern about dengue (from 21% [42/200] to 56% [224/400], indicating they were “very concerned”) and high awareness of key messages, with 81% (324/400) stating they would seek immediate care for severe symptoms. The humour-based approach was rated as appealing or very appealing by 76% (304/400) of respondents, with 90% (360/400) reporting increased likelihood of preventive action.

**Discussion:**

These findings demonstrate that culturally relevant, humour-based RCCE, integrated across digital and traditional media, may be effective in enhancing awareness and shifting perceptions.

## PROBLEM

In early 2024, Lao People's Democratic Republic experienced a sharp increase in reported dengue virus cases, reaching levels comparable to the country’s major 2013 outbreak, which recorded 44 171 cases and 95 deaths. (*1*) This surge highlighted the persistent and serious threat dengue poses to public health in the country and its population of 7.78 million, especially during the tropical monsoon season from June to September, when environmental conditions, such as increased rainfall, create favourable habitats for mosquito breeding and enhance virus transmission. (*2*, *3*)

Although awareness of preventive measures, such as eliminating mosquito breeding sites and protecting against mosquito bites, is relatively high and has been a focus for previous promotions undertaken by the Ministry of Health and provincial and district authorities, survey data suggest these practices are not consistently adopted across communities. (*4*) This gap between knowledge and actual behaviour hampers efforts to reduce mosquito populations and control disease spread. Given dengue’s recurring outbreaks, sustaining preventive actions over time is crucial to minimizing future surges. (*5*)

Effective dengue control requires broad and consistent adoption of critical behaviours, including removing standing water, using personal protective measures and promptly seeking medical care when severe symptoms appear; delays in seeking treatment contribute to dengue fatalities in the country. (*6*) Timely treatment at designated hospitals is essential to prevent severe illness and fatalities. Without ongoing behavioural change supported by community engagement to address these gaps, the country remains vulnerable to repeated outbreaks that strain health-care resources and negatively affect socioeconomic conditions. (*7*)

## CONTEXT

Dengue fever is a mosquito-borne, viral disease endemic to Lao People's Democratic Republic, regularly causing outbreaks. (*1*) The primary vector responsible for transmission is the *Aedes aegypti* mosquito, which thrives in tropical and subtropical environments. The country’s tropical monsoon climate, marked by distinct wet and dry seasons, creates optimal conditions for mosquito breeding, particularly during the rainy season from June to September. (*3*) During this period, increased rainfall results in the widespread accumulation of stagnant water, forming abundant mosquito habitats and elevating the risk of dengue transmission. (*2*, *3*)

Dengue infections can range from mild febrile illness to severe dengue haemorrhagic fever, which can be fatal without proper treatment. (*3*) Several ongoing challenges contribute to the persistent transmission of dengue in the country, including inconsistent community adoption of preventive behaviours and difficulties sustaining effective vector control programmes. Rapid urbanization, population growth and inadequate sanitation further facilitate mosquito breeding and disease transmission. (*5*) Climate change is also expected to worsen the situation. (*1*)

The country has historically experienced cyclical dengue outbreaks every few years, with major epidemics reported in 2013 and 2019. (*7*) These recurrent surges place considerable strain on the country’s public health resources and underscore the need to strengthen prevention and response efforts. As a result, the Ministry of Health, in collaboration with the World Health Organization (WHO) and other partners, has prioritized the enhancement of surveillance systems and case management to reduce fatalities, expanding public education initiatives and implementing innovative strategies to reduce dengue transmission and its overall impact across the nation. (*1*)

## ACTION

**Fig. 1:**
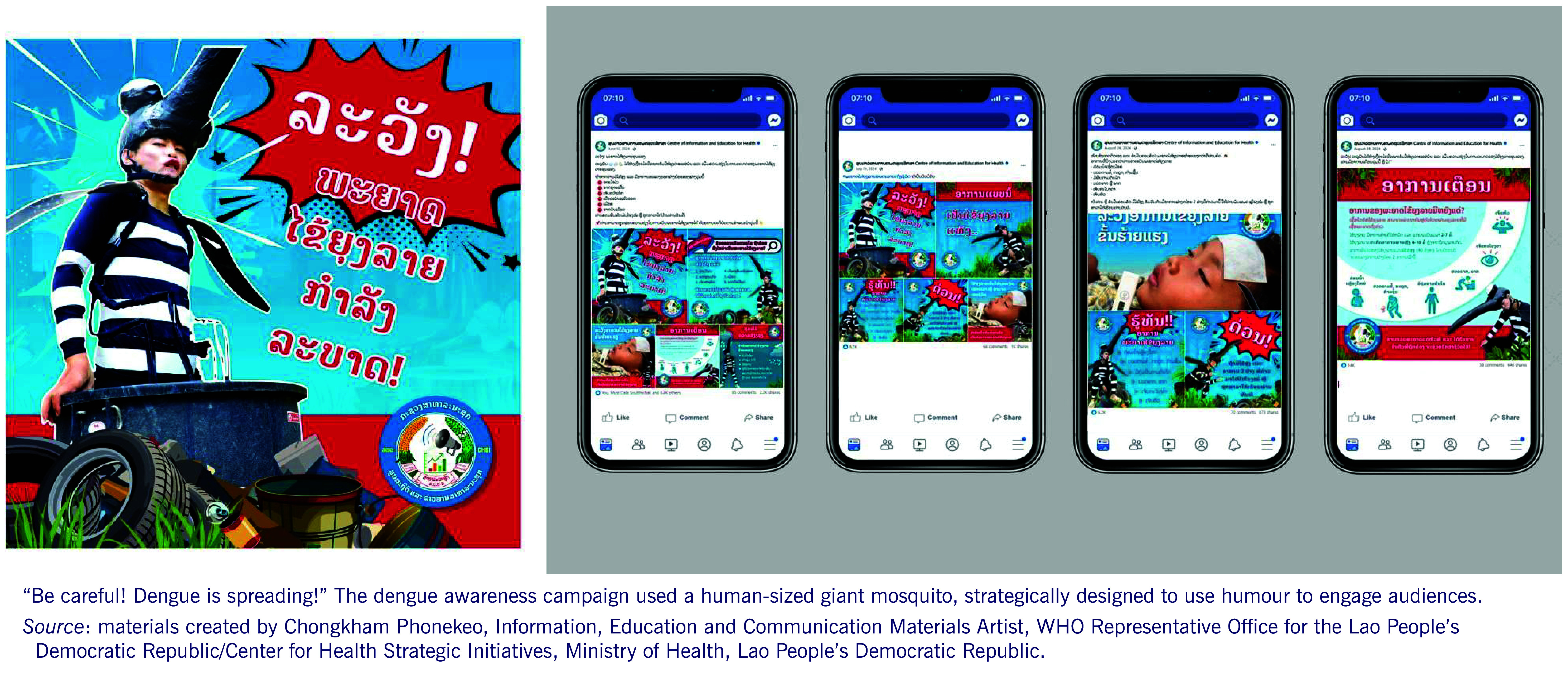
Humour-based images used in the dengue awareness campaign, Lao People’s Democratic Republic, April–September 2024

**Fig. 2:**
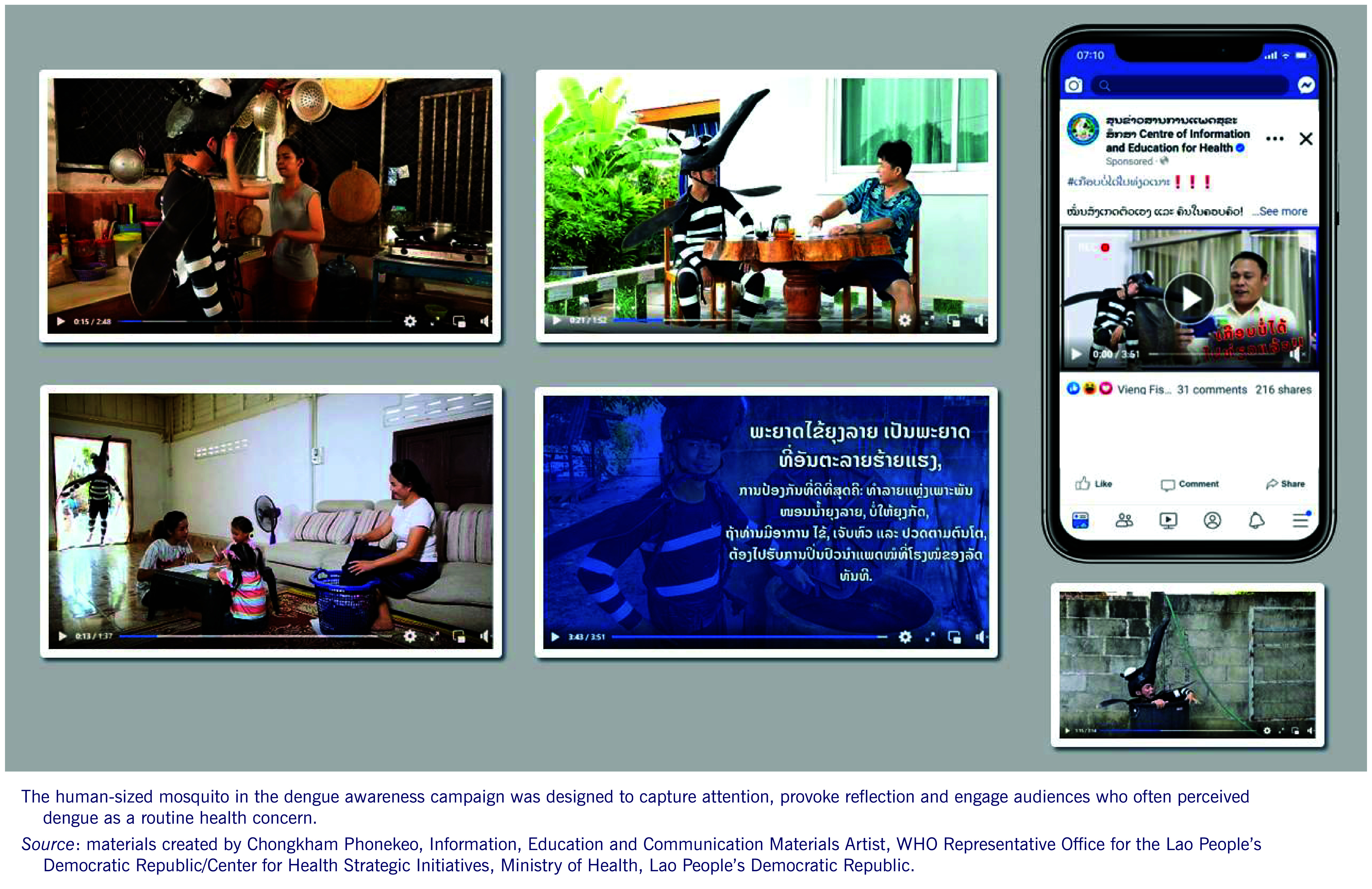
Humour-based videos used in the dengue awareness campaign, Lao People’s Democratic Republic, 2024

In early 2024, to enhance dengue mitigation efforts amid a surge of cases, a humour-driven communication campaign was developed by the Ministry of Health’s Center for Health Strategic Initiatives (CHSI), with technical and financial support from WHO. The strategy particularly targeted the 55% of the population (4.25 million) who use Facebook, the country’s most popular digital platform, while also engaging traditional media and implementing village outreach to include other groups. Central to the campaign was an actor dressed as a mosquito who was portrayed as deliberately and humorously bothersome to people trying to go about their daily activities (**Fig. 1** and **2**). These striking visuals were designed to capture attention and engage social media audiences, and drew on tropes that had been well received during previous campaigns in the country. Conducted from April to September 2024, the campaign specifically targeted individuals who were indifferent to dengue or saw it as a routine health concern (**Fig. 1** and **2**).

[**insert** Figure 1]

[**insert** Figure 2]

Humour was chosen as the primary tool because, although it requires cultural context and alignment, it effectively draws attention and encourages active listening and information retention. (*8*) When people laugh, they become more receptive, enabling important health messages to be communicated in a positive, approachable and non-intimidating way. (*8*) Additionally, leveraging humour through social media was identified by local practitioners as the most cost-effective approach, offering wide reach at relatively low expense.

Incorporating behavioural insights was central to shaping the campaign materials to achieve the greatest possible impact. Public engagement with the humour-based campaign was assessed using a mixed-methods approach to capture both reach and audience response. A nationwide phone survey of 500 respondents was conducted in October 2024 to gather representative insights from diverse geographical and demographic groups. In addition, three nationwide online surveys, each with 1000 respondents, were implemented at key intervals before the campaign launch (July 2023), immediately after its conclusion (September 2024) and during a follow-up phase (October 2024), to track changes in awareness, perceptions and intended behaviours over time. Complementing these surveys, comprehensive social media analytics were employed to quantify content reach, audience interactions and engagement patterns across Facebook.

Campaign materials were pre-tested to ensure resonance with the target audience, including with remote and rural communities. Promotion focused heavily on social media, emphasizing key messages such as mosquito control, symptom recognition and timely referral for severe dengue cases. Complementing digital outreach, the CHSI engaged with traditional media channels, including television and radio, to provide regular advice, warnings, updates on dengue case counts and other relevant information.

**Fig. 3:**
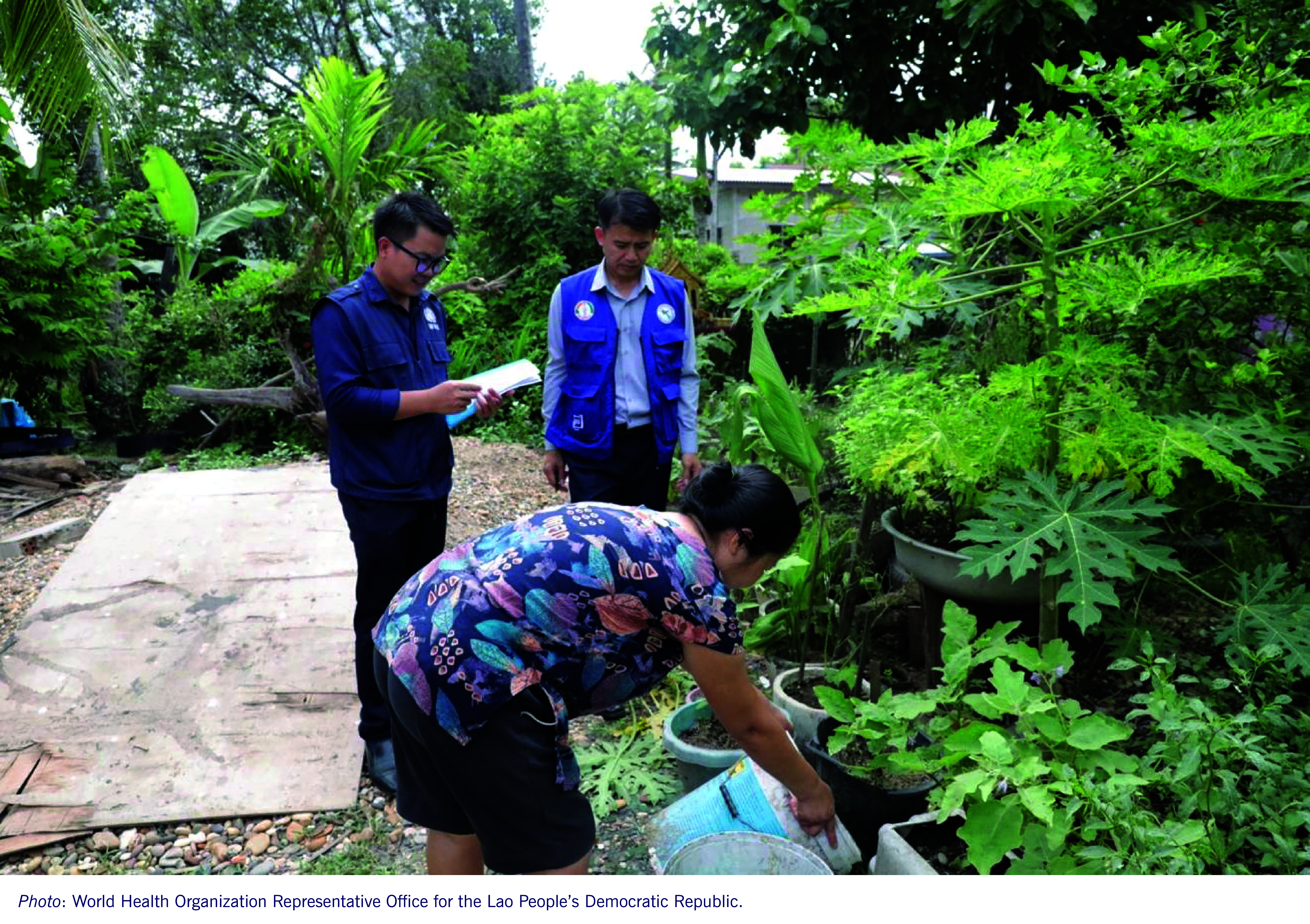
WHO teams visiting communities to better understand knowledge, attitudes and behaviours around vector control, Lao People’s Democratic Republic, 2024

Recognizing that mass media cannot reach every community in the country, the CHSI and WHO undertook community focus group discussions and observational sessions around households in representative communities to identify factors that were successful in encouraging effective behavioural change, such as encouraging more involvement by village authorities and proactive outreach by health centre staff, and related behavioural insights, for potential integration into future outreach (**Fig. 3**).

[**insert** Figure 3]

## OUTCOME

The dengue awareness campaign yielded strong results on social media engagement, public awareness and behavioural intent. Continually updating campaign materials throughout the dengue season proved highly effective in maintaining audience interest. In addition, it provided a strong foundation for informing communication strategies and content development for 2025, with revised and updated materials in use at the time of this publication.

On social media, the campaign achieved extensive reach and engagement. Content was viewed approximately 27 million times by almost 2.9 million unique users (reaching 68.2% of the country’s 4.25 million Facebook users), (*9*) generating more than 86 342 comments, likes and shares. Video content was particularly successful, receiving more than 4.6 million views of at least 15 seconds, including 804 148 long-form views exceeding 60 seconds. These metrics demonstrate the campaign’s ability to capture and sustain public attention across multiple platforms.

Survey data further underscore the campaign’s impact on public perception and awareness. The October 2024 national phone survey revealed that after the campaign, respondents ranked dengue as the country’s most urgent health concern. Similarly, online surveys showed a significant increase in public concern, with the proportion of individuals who reported being “very concerned” about dengue rising from 21% (42/200) before the campaign to 56% (224/400) afterwards.

The campaign also effectively delivered key public health messages and encouraged preventive behaviours. Awareness of critical messaging was high, with 81% (324/400) of respondents indicating they would immediately visit a hospital or health centre if they suspected severe dengue symptoms. Additionally, the humorous giant mosquito theme resonated strongly with audiences: 76% (304/400) of respondents rated the campaign as appealing or very appealing, and 90% (360/400) reported that it increased their likelihood of taking action to prevent dengue transmission. Together, these outcomes demonstrate the campaign’s success in enhancing both people’s awareness of and motivation to engage in protective behaviours against dengue.

The humour-driven risk communication and community engagement (RCCE) campaign was one part of a multicomponent dengue reduction campaign implemented by authorities that included high-level political engagement, vector control activities and enhanced case management. Importantly, epidemiological data showed a significantly flattened curve of dengue case numbers in 2024, although attributing this change to any one or all parts of the government’s dengue response is not possible with the data currently available.

## Discussion

The sharp rise in dengue cases in Lao People's Democratic Republic in early 2024, which reached levels comparable to the major 2013 outbreak, highlights the ongoing challenge dengue poses as a significant public health threat. Despite relatively widespread awareness of preventive measures, their inconsistent practice has contributed to sustained dengue transmission. This gap between knowledge and action emphasizes the urgent need for strategies that effectively motivate behavioural change. These challenges reflect broader trends in endemic regions where environmental factors, such as the tropical monsoon climate and rapid urbanization, create ideal conditions for mosquito breeding and increased disease transmission. (*1*-*5*)

The dengue awareness campaign centred on a humorous giant mosquito and demonstrated the potential of developing innovative communication strategies to engage populations who may be indifferent to or disengaged from dengue prevention. Given the level of indifference to dengue among the intended audience, the use of humour, based on tropes that had been effectively deployed in the country previously, resonated culturally as the campaign reflected everyday life but in comedic ways. Thus, it effectively captured public attention, enhanced message retention and conveyed important health information in a non-threatening manner. This approach aligns with behavioural science insights showing that positive emotional engagement increases receptivity and encourages protective actions. (*8*, *10*) It highlights that humour must be both culturally and contextually aligned to resonate with people, while emphasizing the need for a considered approach to avoid the risk of audience backlash and to ensure that official advice does not undermine the seriousness of the issue.

The campaign’s implementation across multiple platforms including social media, television and radio ensured broad reach and accessibility across diverse demographic groups. Impressive social media metrics, with more than 27 million views and nearly 3 million unique users, combined with strongly positive survey responses, indicated successful public engagement. Notably, the marked increase in public concern about dengue following the campaign signalled an important shift in risk perception, a key driver of protective behaviour.

Furthermore, the campaign successfully translated increased awareness into intentions to adopt preventive measures. A high proportion of respondents indicated that they would promptly seek medical care for severe dengue symptoms, and that the giant mosquito character had strongly resonated with them. Most of the participants rated the campaign as appealing and reported an increased likelihood that they would engage in mosquito control activities. These results suggest enhanced community readiness to address dengue risk effectively, and were reflected by the continuation and expansion of the campaign in 2025. However, additional research is necessary to determine whether there was long-term behavioural change and to confirm whether intention translated into action. (There was limited scope to assess the long-term effects of the 2024 campaign due to the emergency nature of the outbreak.)

Similarly, sustaining behavioural change in endemic settings requires ongoing public engagement. While the survey results were highly positive, with both phone and online survey data aligning, online data were self-reported and sourced from social media users, potentially creating a slight bias towards over-reporting the impact, and highlighting a broader challenge to RCCE in the country’s context. While more than 55% of the population uses Facebook, enabling rapid interaction with compelling content, modern mass communication tools, including TV and radio, remain inaccessible for a substantial proportion of the population, including ethnic minorities living in remote areas. Thus, costly, dedicated outreach in local languages and dialects is required, despite limited resources. The seasonal and recurrent nature of dengue outbreaks calls for the continual monitoring and adaptation of communication strategies to maintain vigilance and prevent message fatigue. Future efforts could benefit from localized messaging tailored to specific communities, expanded outreach efforts and enhanced prioritization of dengue by officials beyond the health sector.

The resolution of structural challenges is critical in complementing communication initiatives, such as through the improvement of health-care access, clinical management approaches and vector control infrastructure. Ensuring that these efforts are addressed in line with the national dengue strategy, under its comprehensive dengue control framework, will equip the country to reduce the disease burden sustainably.

This humour-centred communication campaign provided valuable insights into effective public health messaging in a resource-limited setting where dengue is endemic. By leveraging behavioural insights and combining digital tools with traditional media, the campaign not only raised awareness but also positively influenced public attitudes and behavioural intentions towards dengue prevention. These findings support ongoing innovation and investment in culturally relevant, evidence-based communication strategies as vital elements of successful dengue control programmes.

## References

[1] From the ground up: community-led efforts keeping dengue at bay in Lao PDR [website]. Manila: WHO Regional Office for the Western Pacific; 2025. Available from: https://www.who.int/westernpacific/newsroom/feature-stories/item/from-the-ground-up — community-led-efforts-keeping-dengue-at-bay-in-lao-pdr, accessed 29 April 2026.

[2] Laos [website]. Vientiane: World Mosquito Program; 2025. Available from: https://www.worldmosquitoprogram.org/en/global-progress/laos, accessed 1 October 2025.

[3] Umar M, Asghar S, Zafar S. Environmental and socioeconomic determinants of dengue fever risk in Lao People’s Democratic Republic: A systematic review. J Infect Public Health. 2026 Mar;19(3):103119. 10.1016/j.jiph.2025.103119 41477987

[4] Abdullah A. Urbanization and its influence on public health in Southeast Asia. J Dev Ctry Stud. 2024;8(2):31–43. 10.47604/jdcs.2675

[5] Soukavong M, Thinkhamrop K, Pratumchart K, Soulaphy C, Xangsayarath P, Mayxay M, et al. Bayesian spatio-temporal analysis of dengue transmission in Lao PDR. Sci Rep. 2024 Sep 12;14(1):21327. 10.1038/s41598-024-71807-3 39266587 PMC11393087

[6] Sengkeopraseuth B, Vongphrachanh P, Khamphaphongphane B, Lum L, Luo D, Phengxay M, et al. An investigation of dengue deaths in Vientiane Capital and Champasack Province, Lao People’s Democratic Republic, 2013. J Med Clin Case Rep. 2024;1(12):1–6. 10.61615/JMCCR/2024/NOV027141112

[7] Dengue and severe dengue [website]. Manila: WHO Regional Office for the Western Pacific; 2020. Available from: https://www.who.int/westernpacific/health-topics/dengue, accessed 1 October 2025.

[8] Meyer JC, Venette SJ. Humor in health and risk messaging. In: Powers M, editor. Oxford research encyclopedia of communication. New York (NY). Oxford: Academic; 2017., 10.1093/acrefore/9780190228613.013.507

[9] Kemp S. Digital 2025: Laos. [website]. Singapore: DataReportal; 2025. [cited 2025 Oct 1]. Available from: Available from https://datareportal.com/reports/digital-2025-laos

[10] Miller E, Bergmeier HJ, Blewitt C, O’Connor A, Skouteris H. A systematic review of humour-based strategies for addressing public health priorities. Aust N Z J Public Health. 2021 Dec;45(6):568–77. 10.1111/1753-6405.13142 34411385

